# Hernie de Bochdaleck étranglée: cause rare d’occlusion intestinale aiguë

**DOI:** 10.11604/pamj.2019.34.90.18427

**Published:** 2019-10-16

**Authors:** Mustapha Iken, Adil Mai, Fatimzahra Choukrad, Meryem Haloua, My Youssef Alaoui Lamrani, Meryem Boubbou, Mustapha Maâroufi, Badreedddine Alami

**Affiliations:** 1 Service de Radiologie, Hôpital des Spécialités, CHU Hassan II Fès, Fès, Maroc

**Keywords:** Hernie diaphragmatique congénitale, patient, hernie de Bochdalek à contenu colique étranglée, Congenital diaphragmatic hernia, patient, strangulated Bochdalek’s diaphragmatic hernia

## Abstract

Nous rapportons l'observation d'un jeune homme âgé de 28 ans présentant une hernie diaphragmatique congénitale, qui s'est révélée tardivement par un syndrome occlusif se manifestant sur le scanner par une distension grêlique et colique en amont d'une hernie de Bochdalek à contenu colique étranglée. Le patient a bénéficié d'une intervention chirurgicale par laparotomie avec réduction de la hernie et fermeture de la brèche diaphragmatique. Notre objectif à travers ce document est de mettre le point sur une étiologie rare d'occlusion intestinale qui est la hernie de Bochdalek étranglée, dont le diagnostic reste essentiellement radiologique, basé sur les données du scanner, ce qui permet d' assurer une prise en charge à temps.

## Abstract (en)

We report the case of a 28 year old young man with congenital diaphragmatic hernia, detected at a later stage due to occlusive syndrome manifesting on the scanner as small intestine and colonic distension upstream of a strangulated Bochdalek’s diaphragmatic hernia. The patient underwent laparotomy with reduction of the hernia and closure of the diaphragmatic rupture. This study aims to update the current understanding of a rare cause of intestinal obstruction, strangulated Bochdalek’s diaphragmatic hernia, whose diagnosis is essentially based on radiological examinations based on the scanner data, which allows early management.

## Introduction

L'occlusion intestinale aigüe est une situation fréquente aux urgences, les étiologies sont dominées par la bride au niveau de l'intestin grêle et par la catégorie tumorale au niveau du colon, mais il existe des étiologies rares que le radiologue doit connaitre, la hernie de Bochdalek étranglée, en étant une étiologie de cette catégorie, constitue l'objet de cet article.

## Patient et observation

**Figure 1:**
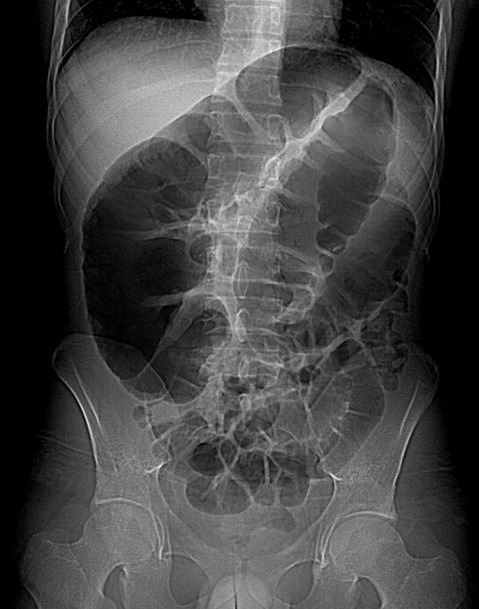
ASP: importante distension colique ainsi que de quelques anses grêliques; on note l´absence de gaz intra-thoracique sur ce liché (caché par l´opacité splénique)

**Figure 2:**
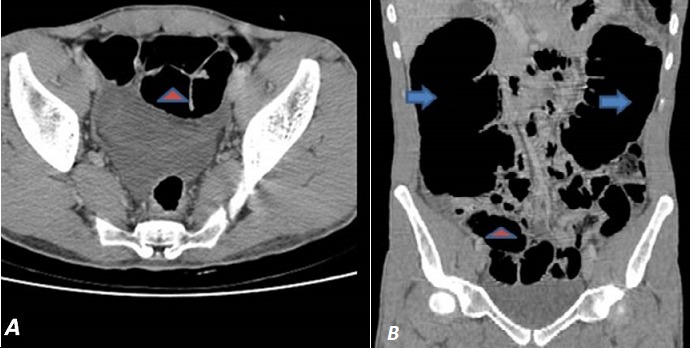
Coupe coronale (A) et coupe axiale (B): montrant la distension colique (flèches bleues) et grêlique (têtes de flèches) à contenu hydro-aérique majoritairement aérique

**Figure 3:**
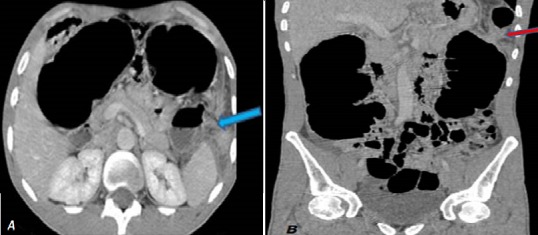
Coupes axiales (A) et coupe coronale (B): montrant la zone de disparité de calibre au niveau d’un défect diaphragmatique postéro-latéral gauche (flèches bleue et rouge)

Il s'agit d'un patient âgé de 28 ans, sans antécédents pathologiques notables, admis aux urgences pour un syndrome occlusif remontant à 4 jours et fait de distension abdominale, d'un arrêt des matières et des gaz et d'une douleur abdominale légère, l'ensemble évoluait dans un contexte d'apyrexie et de conservation de l'état général. L'examen clinique a trouvé un patient conscient, stable sur le plan hémodynamique et respiratoire, avec une légère sensibilité abdominale et un tympanisme. Le toucher rectal a révélé une ampoule rectale vide. Un ASP (Figure 1) réalisé en première intention a objectivé une importante distension colique ainsi que de quelques anses grêliques. Par la suite un scanner abdomino-pelvien avant et après injection du produit de contraste iodé a été réalisé et ayant objectivé une distension colique arrivant à 7,7 cm et grêlique arrivant à 3cm, à contenu hydro-aérique majoritairement aérique (Figure 2), en amont d'une zone de disparité de calibre correspondant à l'étranglement de l'angle colique gauche lors de son passage via un défect diaphragmatique en postéro-latéral gauche (Figure 3). La prise en charge a consisté en une chirurgie urgente sous anesthésie générale par laparotomie médiane à cheval sur l'ombilic, à l'exploration il a été noté une importante distension du caecum, du colon ascendant et du colon transverse, en amont d'une hernie diaphragmatique gauche étranglée, le sac herniaire contenait l'angle colique gauche, le colon descendant et le grand épiploon. Le geste s'est résumé à un élargissement du collet, une réduction du contenu herniaire, une fermeture de la brèche par des points séparés à la soie et un drainage en sous hépatique suivi par la fermeture de la paroi abdominale.

## Discussion

La hernie diaphragmatique congénitale peut se révéler tardivement dans 10 à 30% des cas, posant alors un problème diagnostique [1]. La hernie de Bochdalek (HB) de l'adulte peut rester donc longtemps asymptomatique, avant de provoquer des signes cliniques mixtes, respiratoires par compression du poumon et digestifs par strangulation des viscères comme dans notre observation. Vu la rareté de cette entité chez l'adulte et la non spécificité des signes cliniques, l'imagerie, notamment le scanner, reste le moyen incontournable pour assurer le diagnostic. Sur la radiographie thoracique, une HB peut se traduire par une image hétérogène contenant des opacités et des clartés de siège postéro-latéral, par la présence d'une poche à air gastrique en intrathoracique en cas d'hernie gastrique, ou l'existence d'anses digestives basithoraciques [2]. Cependant, la radiographie standard présente quelques limites, en fait la réduction spontanée de la hernie et l'HB volumineuse qui peut mimer une éventration diaphragmatique peuvent être à l'origine de faux négatifs [3]. L'image basithoracique solido-aérique qui peut simuler une tumeur pulmonaire, pleurale ou diaphragmatique sur le cliché de face, une tumeur médiastinale sur le cliché de profil, la bulle d'emphysème, l'abcès du poumon ou encore la pleuropneumopathie sont des sources de faux positifs. En cas de doute, le transit œsogastroduodénal et le lavement baryté permettent de repérer les viscères creux herniés et le siège du collet herniaire [4]. Mais, sur le transit baryté, la HB peut être confondue avec une hernie hiatale ou avec une éventration diaphragmatique. La TDM reste alors l'examen le plus performant car il permet d'identifier les viscères intra-thoraciques, de préciser le siège du collet herniaire, et plus rarement de découvrir une HB controlatérale [5]. Si elle n'est pas recherchée, la HB peut cependant être méconnue sur la TDM [6]. L'exploration chirurgicale sous cœlioscopie ou par laparotomie peut également méconnaître une HB si le chirurgien n'est pas averti. La symptomatologie clinique polymorphe et non spécifique ainsi que les limites de la radiographie thoracique, expliquent que le diagnostic de HB est souvent posé chez l'adulte à l'occasion des complications aiguës qui peuvent être d'ordre respiratoire ou digestif, en fait le collapsus du poumon provoqué par l'effet de masse des viscères abdominaux passés dans le thorax est à l'origine d'une dyspnée aiguë, Il peut s'y associer un déplacement du médiastin et une diminution du retour veineux cave qui peut entraîner un arrêt cardiaque [7]. Les complications digestives les plus fréquentes sont la strangulation de l'estomac, et la strangulation de l'intestin grêle ou du côlon qui se manifestent par un syndrome occlusif comme le cas de notre patient. Plus rarement, la strangulation du tube digestif peut provoquer une ulcération hémorragique, ou une perforation diastatique ou ischémique [8]. La perforation du tube digestif se manifeste par un pyopneumothorax et un choc septique avec parfois décès brutal [9]. Exceptionnellement, ont été observées des pancréatites aiguës par strangulation du corps du pancréas dans la HB, et des infarctus ou des ruptures spléniques par strangulation du pédicule splénique [9]. En raison de la gravité des complications, la HB doit être systématiquement opérée même si elle est asymptomatique [10]. Le traitement, dans la HB étranglée, consiste en une réduction avec fermeture du défect diaphragmatique et résection des segments non viables en cas de complication ischémique. En l'absence de complications digestives ou pulmonaires, la vidéothoracoscopie et la cœlioscopie peuvent être proposées [9]. La thoracotomie semble plutôt indiquée en cas de complications respiratoires ou de pyothorax permettant ainsi de réduire le contenu herniaire, de laver la cavité pleurale et de refermer la brèche diaphragmatique.

## Conclusion

La HB est une cause rare d'occlusion intestinale chez l'adulte. Le diagnostic doit donc être évoqué devant toute symptomatologie respiratoire ou digestive mal expliquée. La radiographie standard n'est pas suffisamment fiable pour le diagnostic positif de la hernie ni pour son étranglement d'où le rôle majeur le scanner thoraco-abdominal qui permet éventuellement la mise en évidence d'une complication digestive nécessitant une intervention en urgence.

## Conflits d’intérêts

Les auteurs ne déclarent aucun conflit d'intérêts.

## References

[1] Cherifi A, Ferrouk O, Boudiaf L, Bellamine (2015). Un cas de hernie de Bochdalek à révélation tardive.

[2] Zenda T, Kaisaki C, Mori Y, Miyamoto S, Horichi Y, Nakashima A (2000). Adult right-sided Bochdalek hernia facilitated by coexistent hepatic hypoplasia. Abdom Imaging.

[3] Bujanda L, Larrucea I, Ramos F, Munoz C, Sanchez A, Fernandez I (2001). Bochdalek's hernia in adults. J Clin Gastroenterol.

[4] Shah S (2000). Laparoscopic repair of a chronic diaphragmatic hernia. Surg Laparosc Endosc Percut Tech.

[5] Wyler S, Muff B, Neff U (2000). Laparoskopischer Verschluss einer Bochdalek-Hernie Beim Erwachsenen. Chirurg.

[6] Steenhuis LH, Tjon A, Tham RTO, Smeenk FWJM (1994). Bochdalek hernia: a rare cause of pleural empyema. Eur Respir J.

[7] Salaçin S, Alper B, Cekin N, Gülmen MK (1994). Bochdalek hernia in adulthood: a review and an autopsy case report. J Forensic Sci JFSCA.

[8] Tzeng JJ, Lai KH, Lo GH, Hsu JH, Mok KY (2001). Gastropleural fistula caused by incarcerated diaphragmatic herniation of the stomach. Gastrointest Endosc.

[9] Habib E, Bellaïche G, Elhadad A (2002). Complications de la hernie de Bochdalek méconnue de l'adulte. Ann Chir.

[10] Schwartz A, Dozolneux G, Desjardin M, Evrard S, Bechade D (2013). Hernie diaphragmatique symptomatique à distance d'une ablathérapie pulmonaire par radiofréquence. Journal de chirurgie viscérale.

